# Susceptibility of *Leishmania amazonensis* Axenic Amastigotes to the Calpain Inhibitor MDL28170

**DOI:** 10.3390/tropicalmed9110259

**Published:** 2024-10-31

**Authors:** Simone S. C. Oliveira, Fernanda A. Marinho, Leandro S. Sangenito, Sergio H. Seabra, Rubem F. Menna-Barreto, Claudia M. d’Avila, André L. S. Santos, Marta H. Branquinha

**Affiliations:** 1 Laboratório de Estudos Avançados de Microrganismos Emergentes e Resistentes (LEAMER), Departamento de Microbiologia Geral, Instituto de Microbiologia Paulo de Góes (IMPG), Universidade Federal do Rio de Janeiro (UFRJ), Rio de Janeiro 21941-902, Brazil; simonesantiagorj@yahoo.com.br (S.S.C.O.); fernandamarinho.bio@gmail.com (F.A.M.); leandro.sangenito@ifrj.edu.br (L.S.S.); 2 Instituto Federal de Educação, Ciência e Tecnologia do Rio de Janeiro, Campus Nilópolis, Rio de Janeiro 26530-060, Brazil; 3 Laboratório de Biologia Celular e Tecidual, Universidade Estadual do Norte Fluminense Darcy Ribeiro, Campos dos Goytacazes, Rio de Janeiro 28013-602, Brazil; seabrash@uenf.br; 4 Laboratório de Biologia Celular, Instituto Oswaldo Cruz (IOC), Fundação Oswaldo Cruz (FIOCRUZ), Rio de Janeiro 21040-360, Brazil; rubemb@ioc.fiocruz.br; 5 Laboratório de Doenças Parasitárias, Instituto Oswaldo Cruz (IOC), Fundação Oswaldo Cruz (FIOCRUZ), Rio de Janeiro 21040-360, Brazil; claudia.davila@ioc.fiocruz.br; 6 Programa de Pós-Graduação em Bioquímica, Instituto de Química, Universidade Federal do Rio de Janeiro (UFRJ), Rio de Janeiro 21941-909, Brazil

**Keywords:** *Leishmania*, axenic amastigotes, calpain inhibitor, alternative chemotherapy

## Abstract

Leishmaniasis encompasses a group of neglected diseases caused by flagellated protozoa belonging to the *Leishmania* genus, associated with high morbidity and mortality. The search for compounds with anti-*Leishmania* activity that exhibit lower toxicity and can overcome the emergence of resistant strains remains a significant goal. In this context, the calpain inhibitor MDL28170 has previously demonstrated deleterious effects against promastigote forms of *Leishmania amazonensis*, which led us to investigate its role on axenic amastigote forms. The calpain inhibitor MDL28170 was able to decrease the viability of amastigotes in a typically dose-dependent manner. The treatment with the IC_50_ dose (13.5 μM) for 72 h led to significant amastigote lysis and increased cell-to-cell aggregation. Ultrastructural analysis revealed several cellular alterations, including disruption of the *trans*-Golgi network and the formation of autophagosomes when treated with MDL28170 at ½ × IC_50_ dose. Additionally, mitochondrial swelling and the formation of concentric membranous structures inside the mitochondrion were observed after incubation with the IC_50_ dose. These results reinforce the potential application of the calpain inhibitor MDL28170 against *L. amazonensis*, highlighting its effectiveness and possible mechanism of action against the parasite.

## 1. Introduction

Leishmaniasis is classified as a group of tropical, neglected, and emerging diseases, frequently associated with factors like malnutrition and lack of financial resources [1,2]. The disease affects mainly some of the poorest nations, being endemic in 98 countries with an annual incidence of 700,000 to 1 million new cases [2]. Leishmaniasis is caused by a heterogeneous group of protozoa belonging to the genus *Leishmania* and may present different clinical manifestations, such as the cutaneous, mucocutaneous, and visceral forms of the disease [3]. The chemotherapy to combat these infections comprises a limited arsenal of drugs such as sodium stibogluconate, meglumine antimoniate, amphotericin B, and miltefosine. However, these drugs present a high cost and have different side effects, besides the emergence of several cases of resistance to these antimicrobials. In this sense, the search for new chemotherapeutic agents is essential to ensure the continued control of this disease [4,5,6].

The presence of a large and diverse family of calpain-related genes in trypanosomatids including *Trypanosoma brucei*, *Trypanosoma cruzi*, some *Leishmania* species, and monoxenic genera called the attention of researchers in the last decades [7,8]. Calpains were originally studied in a diverse range of biological processes in humans, and as the upregulation of several members of the calpain family is involved in human diseases, this family of peptidases has an important therapeutic potential, and a huge effort has been made in the field of research to develop a means of identifying selective calpain inhibitors [9]. In this regard, our research group started to study the effects of the calpain inhibitor MDL28170, which is a potent and cell-permeable inhibitor [10], in *Leishmania amazonensis*, one of the causative agents of human cutaneous leishmaniasis in the Amazon region, Brazil. MDL28170 was added to replicating promastigotes and our results showed that this molecule arrested the growth of these parasites in a dose-dependent manner, besides inducing the expression of apoptotic markers [11,12]. In addition, the calpain inhibitor was able to reduce the interaction process of *L. amazonensis* promastigotes with murine peritoneal macrophages, as well as the infection rate [13]. Subsequently, the susceptibility of six *Leishmania* species (*L. amazonensis*, *L. braziliensis*, *L. major*, *L. mexicana*, *L. infantum*, and *L. donovani*) to MDL28170 was compared. The calpain inhibitor was able to reduce promastigote proliferation and viability as well as the number of intracellular amastigotes in RAW macrophages [14].

As cited above, the development of alternative options to treat leishmaniasis must guarantee the action in both morphological stages of the parasite: promastigotes, which live in the midgut of the sand-fly invertebrate host through which the parasites are transmitted to the vertebrate host, and amastigotes that live in the latter, usually infecting macrophages and responsible for the spreading of the infection in the body [1,6]. Promastigote forms can be easily cultured in axenic culture media such as Schneider’s insect medium, originally designed for the cultivation of insect cell lines by mimicking the physiological properties of insect fluids [15]. On the other hand, amastigote forms are more difficult to maintain in vitro, because they require macrophages as host cells to be cultured [1,16]. In this sense, the cultivation of promastigotes in conditions that mimic the intracellular milieu, like acidic pH and elevated temperature, allows the differentiation of promastigote forms to amastigote forms. The advent of successful in vitro culture of axenic amastigotes permits the quick and easy primary screening of candidate antileishmanial compounds, as performed for promastigotes, but representative of the situation in vivo, since it uses the clinically relevant amastigote stage without the labor-intensive and expensive use of intracellular amastigotes derived from infected macrophages or animal models [17]. In addition to drug screening, axenic amastigotes can be useful to improve comprehension of the mechanism of action of new drug candidates without interference of mechanisms dependent of macrophage activation [18,19,20,21,22]. We have previously determined that the calpain inhibitor MDL28170 reduced the number of intracellular amastigotes in infected macrophages in a clear time- and dose-dependent manner with no increase in nitric oxid (NO) levels, although TNF-α production was positively modulated [13]. In this aspect, the axenic amastigote model has been explored to understand cellular structure, metabolic pathways, and signaling networks of *Leishmania* spp., contributing to the elucidation of several aspects of the parasite life cycle [18,19,22].

There are remarkable differences previously determined between promastigotes and amastigotes with respect to biochemical functions, such as mitochondrial bioenergetics [6,23], which demands the proper investigation of the antileishmanial activity of MDL28170 on extracellular amastigote forms. With this idea in mind, the aim of the present study was to determine the viability and ultrastructural changes after MDL28170 treatment in *L. amazonensis* axenic amastigote forms.

## 2. Materials and Methods

### 2.1. Chemicals

The calpain inhibitor MDL28170 (a reversible peptidomimetic inhibitor, also known as calpain inhibitor III; Z-Val-Phe-CHO; Z = *N*-benzyloxycarbonyl), dimethylsulfoxide (DMSO), Schneider’s insect medium, media constituents, buffer components, and reagents were purchased from Sigma-Aldrich Chemical Co. (St. Louis, MO, USA). Fetal bovine serum (FBS) was obtained from Gibco Life Technology (New York, NY, USA). All other reagents were of analytical grade.

### 2.2. Parasite and Cultivation

*Leishmania amazonensis* (IFLA/BR/1967/PH8 strain) was provided by Coleção de *Leishmania* from Instituto Oswaldo Cruz (*Leishmania* Type Culture Collection-LTTC-WDCM 731). Promastigotes were maintained via weekly transfers in 25 cm^2^ culture flasks with Schneider’s insect medium, pH 7.0, supplemented with 10% FBS and gentamicin 40 µg/mL at 26 °C.

Promastigote-to-amastigote differentiation in a host cell-free culture was carried out as follows: promastigotes in the stationary phase of growth (72 h) were collected, washed 3 times with cold phosphate-buffered saline (PBS; 150 mM NaCl, 20 mM phosphate buffer, pH 7.2) at 3000× *g*, for 5 min at 4 °C, and then transferred (10^7^ cells/mL) to Schneider’s medium at pH 5.5 supplemented with 2.5% FBS and incubated at 32 °C for 6 days [10]. Cells were counted daily in a Neubauer chamber and the number of motile, elongated promastigote forms containing a long flagellum and nonmotile, ovoid amastigote forms with no visible flagellum was estimated by using these morphological criteria. To ascertain the generation of axenic amastigotes, daily aliquots containing 10^7^ parasites were also collected during the differentiation process, washed in PBS, and fixed for 30 min at room temperature in 0.4% paraformaldehyde. After fixation, cells were washed three times in PBS, and the parasites were incubated for 1 h with the promastigote-specific 3A1-La monoclonal antibody diluted at 1:100 [24], kindly donated by Dra. Elvira Saraiva, Institute of Microbiology Paulo de Góes, UFRJ, Brazil. Cells were then extensively washed in PBS and incubated with rabbit anti-IgG secondary antibody conjugated with fluorescein isothiocyanate (FITC) diluted at 1:200. Finally, cells were washed and analyzed via flow cytometry (FACSCalibur, BD Biosciences, San Jose, CA, USA). Non-treated cells and those treated only with the secondary antibody were used as negative controls, while PH8 strain promastigote forms were used as a positive control.

### 2.3. Effect of MDL28170 on Axenic Amastigotes Viability

Axenic amastigote forms (5 × 10^5^ cells/mL) were incubated in 96-well plates at 32 °C for 24, 48, and 72 h with MDL28170 at 5, 10, 15, 20, 25, and 30 µM (starting from a stock solution at 5 mM). The DMSO volume used to prepare the highest concentration of the calpain inhibitor was used as control of the solvent. The viability of axenic amastigotes was checked by using AlamarBlue^®^ [25], while sodium azide (0.01%) was used as a positive control. The determination of the IC_50_ value (the 50% inhibitory concentration) for axenic amastigotes was performed every 24 h of contact with the drug. This value was assessed via linear regression analysis, by plotting the log number of amastigotes versus drug concentration by use of Origin Pro 7.5 computer software.

### 2.4. Effect of MDL28170 on the Ultrastructure of Axenic Amastigotes

Axenic amastigote forms (5 × 10^5^ cells/mL) were treated with the ½ × IC_50_ and IC_50_ doses of MDL28170 for 72 h at 32 °C. Cells were then fixed with 2.5% glutaraldehyde in 0.1 M Na-cacodylate buffer (pH 7.2) at room temperature (25 °C) for 40 min and post-fixed with a solution of 1% OsO_4_, 0.8% potassium ferricyanide, and 2.5 mM CaCl_2_ in the same buffer for 30 min at 25 °C. The cells were dehydrated in an ascending acetone series and embedded in PolyBed 812 resin. Ultrathin sections were stained with uranyl acetate and lead citrate; these sections were examined under a Jeol JEM1011 transmission electron microscope (Jeol Ltd., Tokyo, Japan) at Plataforma de Microscopia Eletrônica, FIOCRUZ. Alternatively, the parasites were dried via the critical point method with CO_2_, mounted on aluminum stubs, coated with a 20 nm-thick gold layer, and examined under a Jeol JSM 6390LV scanning electron microscope.

### 2.5. Statistics

All experiments were performed in triplicate, in three independent experimental sets. The data were analyzed statistically by means of Student’s *t* test using EPI-INFO 6.04 (Database and Statistics Program for Public Health) computer software. *p* values of 0.05 or less were considered statistically significant.

## 3. Results

### 3.1. Generation of L. amazonensis Axenic Amastigote Forms

**Figure 1:**
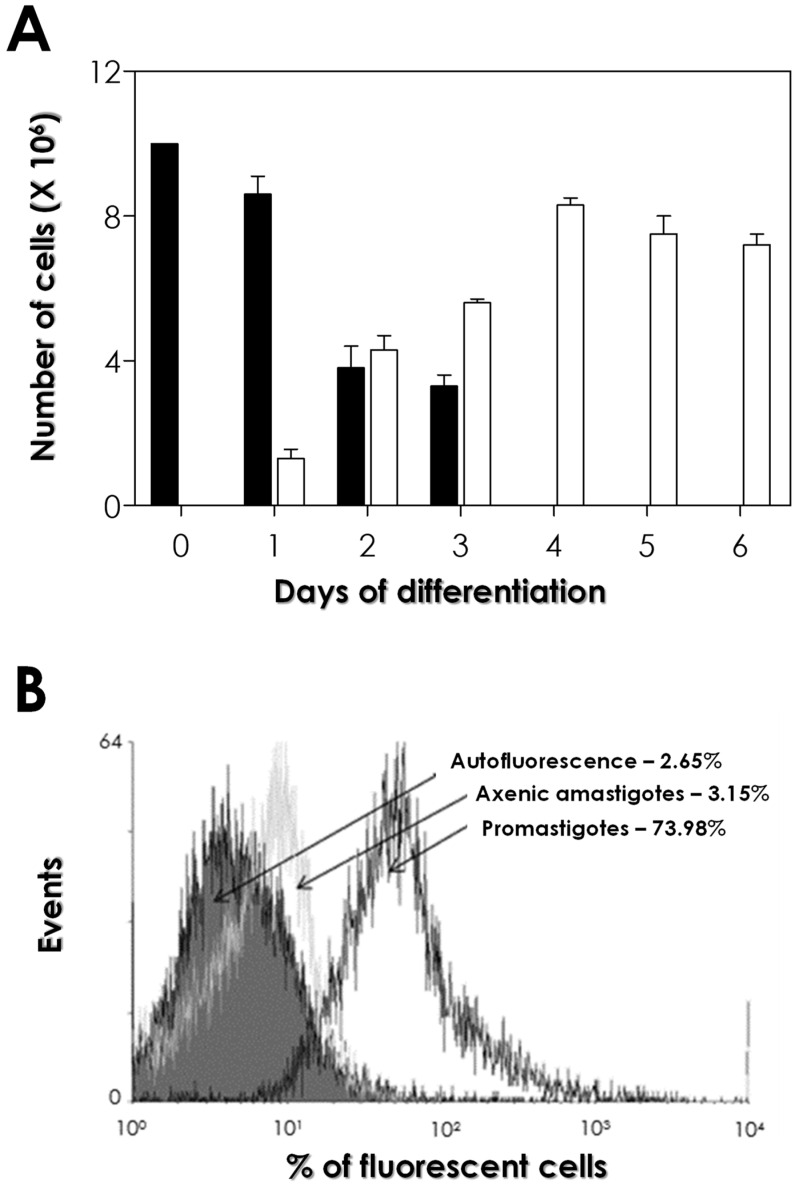
*Leishmania amazonensis* promastigote-to-amastigote differentiation in axenic conditions. (**A**) PH8 strain promastigote forms (10^7^ cells/mL) in stationary phase of growth were incubated at 32 °C in Schneider’s medium at pH 5.5, supplemented with 2.5% FBS. The differentiation process was accompanied for 6 days, and aliquots were taken daily for the quantification of promastigote forms (black bars) and amastigote forms (white bars) in a Neubauer chamber, using cell morphology as the major criterion. The results represent the mean and standard deviation of three independent experiments. (**B**) Axenic amastigote forms (obtained on the 6th day of the differentiation process) and promastigotes in the stationary phase of growth were fixed with paraformaldehyde and incubated in the absence (autofluorescence) or in the presence of promastigote-specific 3A1-La antibody at 1:100 dilution and then analyzed with flow cytometry. The values in parentheses represent the percentage of fluorescent cells. When treated only with the secondary-FITC antibody, cells generated similar curves to that observed in the autofluorescence of cells Representative data of the analysis of 10,000 cells from 1 of 3 experiments are shown.

Figure 1A shows the kinetics of promastigote-to-amastigote differentiation during 6 days of incubation at 32 °C in Schneider’s medium at pH 5.5 containing 2.5% FBS. The results showed that the number of promastigotes started to decrease after 24 h, and these forms were detected up to 72 h. On the other hand, the number of amastigote-like forms increased in a time-dependent fashion, starting after the first day of culture. On the 4th day, most of the cells in the culture corresponded to axenic amastigote-like forms, and this profile remained until the 6th day (Figure 1A).

To confirm the generation of axenic amastigotes, the expression of promastigote-specific molecules was evaluated along the differentiation process using 3A1-La antibody [24]. Promastigotes and axenic amastigote-like forms (the latter obtained on the 6th day of the differentiation process) were washed, fixed in paraformaldehyde, incubated in the presence of 3A1-La antibody, and analyzed via flow cytometry. Promastigote forms exhibited a higher expression of epitopes recognized with the 3A1-La antibody, with an elevated percentage of fluorescent cells (73.98%) when compared to axenic amastigote-like forms, which presented a significantly reduced percentage of fluorescent cells (3.15%) (Figure 1B). The latter value was similar to the autofluorescence of cells, which was 2.65% (Figure 1B). These data, together with those presented in Figure 1A, provide strong evidence that the cells obtained from the differentiation process are in fact axenic amastigote forms. Cells obtained on the 6th day of the differentiation process were then considered axenic amastigote forms and used in the subsequent experiments.

### 3.2. Effects of MDL28170 on the Viability of L. amazonensis Axenic Amastigotes

**Figure 2:**
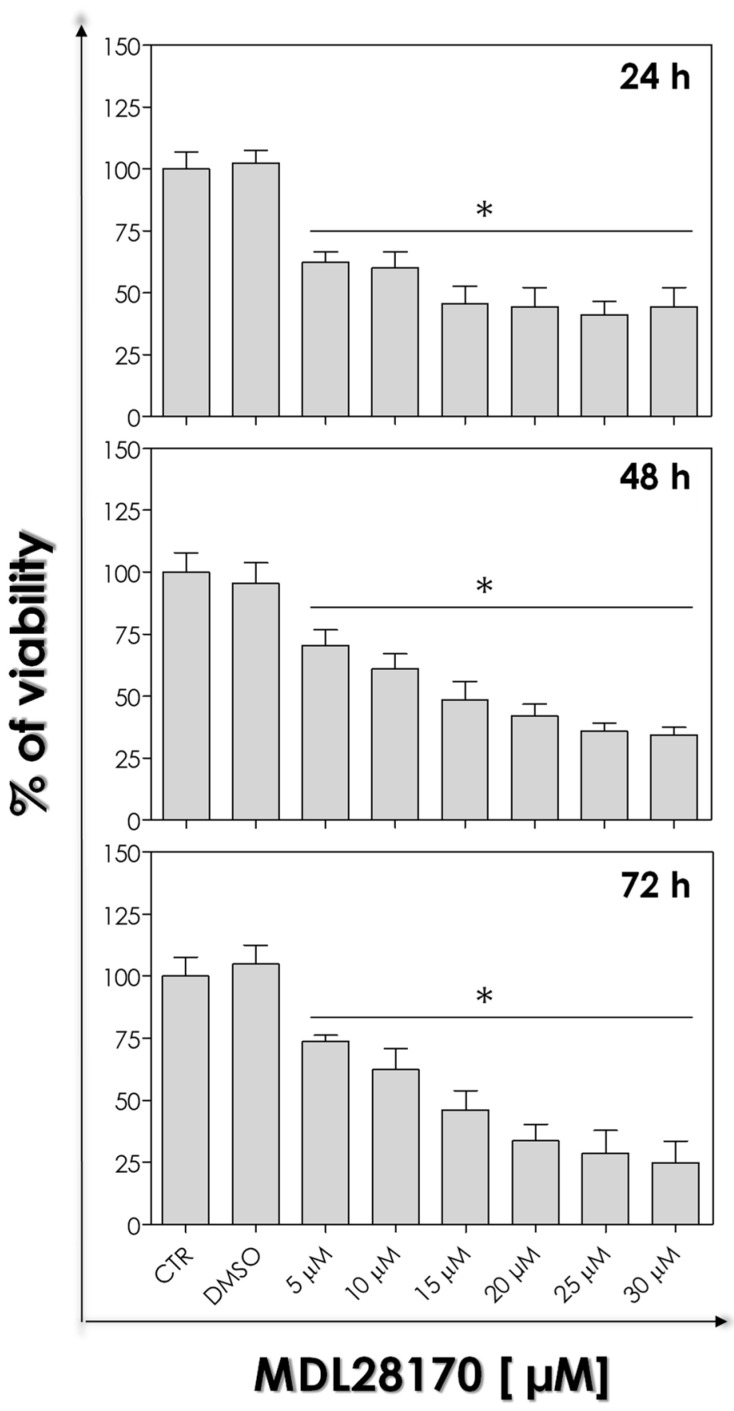
Effect of MDL28170 in the cell viability of axenic amastigote forms of *Leishmania amazonensis*. The viability of amastigotes was performed in the absence (control, CTR) or in the presence of the calpain inhibitor at concentrations ranging from 5 to 30 μM during 24 h, 48 h, and 96 h. The dilution of DMSO used to prepare the highest concentration of the calpain inhibitor was used as a control of the solvent (DMSO). Cells were subsequently incubated with AlamarBlue^®^ as described in the Materials and Methods. Cell viability was expressed as the percentage in comparison to control cells (100%). The asterisks denote the significant differences of axenic amastigotes viability after MDL28170 treatment when compared to non-treated cells. The results represent the mean and standard deviation of three independent experiments (*p* < 0.05).

The cytotoxicity of the calpain inhibitor MDL28170 against axenic amastigote forms was tested with the AlamarBlue^®^ colorimetric assay. Amastigotes were incubated for 24 h, 48 h, and 72 h (Figure 2) in the absence or in the presence of different concentrations of MDL28170 (5, 10, 15, 20, 25, and 30 µM). The calpain inhibitor reduced the cell viability in a dose-dependent manner, but not a time-dependent manner (Figure 2), since the lowest concentration of MDL28170 (5 µM) was able to reduce significantly cell viability after 24 h, and this profile was kept up to 72 h. The IC_50_ values calculated after 24, 48, and 72 h were determined as 17.6 µM, 16.5 µM, and 13.5 µM, respectively. DMSO, the solvent used in the calpain inhibitor solution, did not affect axenic forms viability. Parasites treated with sodium azide were used as positive control of non-viable cells.

### 3.3. Effects of MDL28170 on L. amazonensis Axenic Amastigotes Ultrastructure

**Figure 3:**
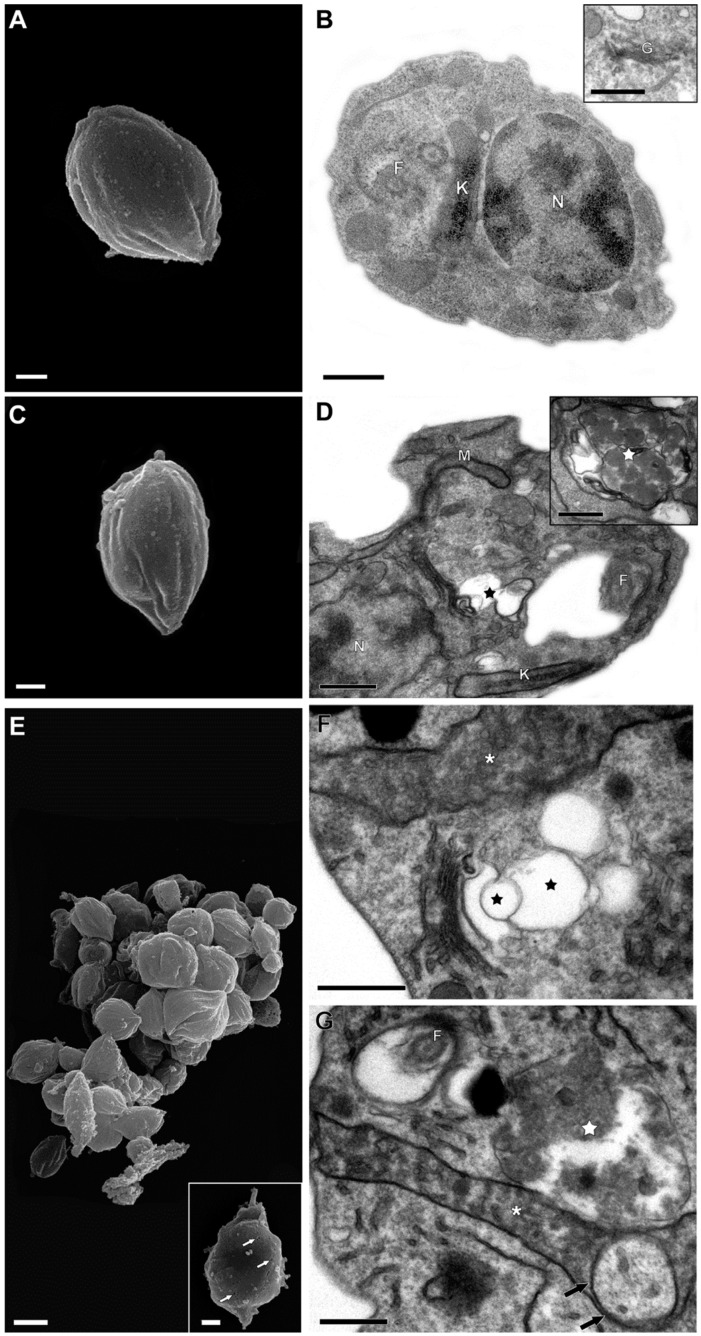
Ultrastructural effects of MDL28170 on *Leishmania amazonensis* axenic amastigotes. Scanning (**A**,**C**,**E**) and transmission (**B**,**D**,**F**,**G**) electron microscopy analyses. (**A**,**B**) Control parasites showed typical morphology of a protozoa body (**A**) and organelles such as a nucleus (N), kinetoplast (K), flagellum (F), and Golgi (G). (**C**,**D**) The treatment with the ½ × IC_50_ dose of MDL28170 led to disruption of *trans*-Golgi network (black star) and the formation of autophagosomes (white star); however, no alterations were observed in amastigotes’ body architecture (**C**). (**E**–**G**) The dose of the IC_50_ value of the inhibitor induced a mitochondrial swelling with loss in the electron density of this organelle matrix (white asterisks), and also the formation of concentric membranar structures inside the mitochondrion (black arrows), as well as Golgi disruption (black stars) and the appearance of autophagosomes (white star) previously detected in parasites treated with the lower concentration of MDL28170. (**E**) Scanning electron microscopy analysis demonstrated an intense amastigotes lysis, better evidenced in the inset (white arrows). Nucleus (N), mitochondrion (M), kinetoplast (K), flagellum (F), and Golgi (G). All bars = 0.5 µm, except by E (bar = 2 µm).

The possible effects of the calpain inhibitor on axenic amastigotes ultrastructure were investigated via both scanning and transmission electron microscopies. For this, parasites were incubated in the absence (control) or in the presence of MDL28170 at ½ × IC_50_ and IC_50_ doses for 72 h. Scanning electron microscopy analysis showed that control parasites presented a typical amastigote morphology, with a rounded cell body and no external flagellum (Figure 3A). Amastigotes incubated in the presence of the ½ × IC_50_ dose of the calpain inhibitor (Figure 3C) displayed a similar morphology to the untreated control cells, while incubation with the IC_50_ dose of MDL28170 led to an intense amastigote lysis, besides a remarkable cell aggregation (Figure 3E).

Transmission electron microscopy analysis revealed that control axenic amastigotes displayed intact cellular structures, such as a nucleus, kinetoplast, flagellum, and Golgi complex (Figure 3B). Parasite cells incubated in the presence of the ½ × IC_50_ dose of MDL28170 showed the disruption of *trans*-Golgi network and the formation of autophagosomes (Figure 3D) as major differences to control cells. Axenic amastigotes incubated with the IC_50_ value of the calpain inhibitor presented similar morphological changes previously detected in parasites treated with the lower concentration of MDL28170 (Figure 3F,G), besides the detection of mitochondrial swelling with a loss of electron density of the organelle matrix and also the formation of concentric membranar structures inside the mitochondrion (Figure 3F,G).

## 4. Discussion

The biological effects of the calpain inhibitor MDL28170 previously determined by our group in the promastigote and intracellular amastigote forms of *L. amazonensis* [12,13,14] encouraged us to evaluate the effect of this drug directly in the viability and ultrastructure of the axenic amastigote forms. In this sense, the in vitro cultivation of amastigote forms has the potential to provide an excellent and limitless source of viable organisms that are free from contaminating host-derived components and can be effectively used in drug evaluation, molecular cloning, identification of new developmentally regulated genes, and vaccine production [26]. Different studies have reported similarities between axenic and intracellular amastigotes in terms of morphology, gene regulation, and enzyme profile; the sensitivity to some reference drugs indicated that the use of axenic amastigotes stands out as an excellent model for the study of alternative chemotherapy options [17,27,28].

Axenic amastigotes have been successfully obtained for various strains of *Leishmania* through temperature elevation as a trigger, either independently or in conjunction with a decrease in pH [29,30]. In the present study, the differentiation protocol for obtaining axenic amastigote forms was based on the acidification of the medium (pH 5.5), reduced serum concentration (2.5%), and elevated temperature (32 °C), starting from a culture of stationary-phase promastigotes [16]. However, conditions that trigger the transformation of promastigotes to amastigotes in one species might not be appropriate for another [26]. Herein, the process of differentiation of promastigote forms into amastigote-like forms was confirmed via analysis of the expression of *L. amazonensis* promastigote-specific epitopes recognized with the monoclonal antibody 3A1-La [24,31]. Our results showed that the promastigote forms exhibited a much greater expression of epitopes recognized with 3A1-La antibody than the differentiated amastigote forms.

The validation of the protocol for obtaining axenic amastigote forms led us to investigate the action of MDL28170 on this evolutive form. Previous studies have shown significant differences in the in vitro susceptibility of promastigotes and amastigotes for the reference drugs [5,6,16]. For this, we verified the viability of axenic amastigote forms using the colorimetric assay Alamarblue^®^. Our results demonstrated a reduction in the viability via incubation with the calpain inhibitor, and the IC_50_ calculated for axenic amastigote forms after 72 h was equivalent to 13.5 µM. Previously, the IC_50_ value calculated for promastigote forms of *L. amazonensis* (Josefa strain) after 72 h [12] was determined as 15 µM. This result demonstrates that the anti-leishmanial activity of MDL28170 is considerable against both axenic amastigotes and promastigotes, displaying similar IC_50_ values in the same time interval. In another work from our group, MDL28170 was also able to significantly reduce the infection rate of murine peritoneal macrophages after 24 h, presenting an IC_50_ value of 18.2 µM for *L. amazonensis* intracellular amastigotes [13]. In addition, RAW macrophages that were infected with six different species of *Leishmania* and then treated with MDL28170 presented a reduction in the infection of all the species studied: the susceptibility of intracellular amastigote forms to MDL28170 was similar to the promastigotes’ IC_50_ values and in the range of low micromolar concentration for all *Leishmania* species tested [14]. In this sense, the IC_50_ values for intracellular amastigotes varied between 2.8 µM and 8.5 µM. It is noteworthy that MDL28170 is considerably more toxic to the parasites than to host cells (CC_50_ value equal to 111.5 µM) [14]. The high selectivity index values calculated suggest that MDL28170 is well tolerated by mammalian cells in comparison to the effects displayed in *Leishmania* spp. [14]. However, an in vitro assessment of the potential toxicity to human phagocytes will be necessary to establish this calpain inhibitor as a viable candidate for the treatment of leishmaniasis.

After evidencing that MDL28170 treatment induced loss of parasite viability, we decided to investigate the effects of this calpain inhibitor on the ultrastructure of MDL28170-treated parasites. In scanning electron microscopy, axenic amastigotes treated with the IC_50_ dose of MDL28170 presented a tendency to form cell aggregates and an intense cell lysis, which confirmed the reduction in cell viability detected via the AlamarBlue^®^ colorimetric assay. These alterations were similar to those reported for *L. amazonensis* promastigote forms, for which the pre-treatment with the IC_50_ value of MDL28170 also led to cell aggregation, and some of the cells that were clumped together presented morphological alterations and cell debris [12].

The ultrastructural changes caused by the calpain inhibitor in axenic amastigotes were visualized via transmission electron microscopy at two distinct concentrations in order to establish which sequence of events may occur. In cells incubated in the presence of the ½ × IC_50_ dose of MDL28170, the disruption of *trans*-Golgi network and the formation of autophagosomes were the prominent alterations detected. After treatment with the IC_50_ dose of the calpain inhibitor, the major alterations detected were mitochondrial swelling with a loss of electron density and the formation of concentric membranar structures inside this organelle. These alterations are quite different from the ones described for promastigotes forms, as discussed below.

When *L. amazonensis* promastigote forms were treated during the same time interval with MDL28170 at the IC_50_ dose [12], the ultrastructural images showed (i) an intense vacuolization in the cytoplasm, (ii) the disorganization of the endocytic pathway, which was found distended and presented reduced electron density, and (iii) the accumulation of small vesicles characteristic of the multivesicular body network. Interestingly, the promastigote mitochondrion displayed a normal shape and density under the same conditions; since a reduction in the mitochondrial transmembrane electric potential was detected when the 2 × IC_50_ value was employed [12], it was suggested that MDL28170 displayed a secondary effect on this organelle in promastigotes. However, different biochemical approaches pointed to the action of MDL28170 as an inducer of an apoptotic-like process in *L. amazonensis* promastigotes [12]. The detection herein of mitochondrial swelling and the presence of concentric membranar structures in the axenic amastigote mitochondrion after treatment with the IC_50_ dose of the calpain inhibitor confirm that this organelle is a crucial target to the action of this drug, which may trigger an autophagic process. However, the mitochondrion is part of a convergent target, and the modifications promoted should be interpreted with caution [32,33].

Evidence indicates that the mitochondrial dysfunction is a critical intracellular signal to induce the autophagic machinery, a process that is triggered by several anti-trypanosomatid compounds [33,34]. In the present work, another feature suggestive of autophagy as the mode of action of MDL28170 in axenic amastigotes was the formation of autophagosomes. Autophagy is a catabolic process that consists of lysosomal degradation of the cytoplasmic content, which is directed into vesicles, the autophagosomes. This mechanism is linked to cell survival under stress conditions, thereby allowing for adaptive protein synthesis, but it can result in cell death when over-activated, such as by drug stimulus [34]. *Leishmania* autophagy has been clearly demonstrated during starvation or cell differentiation [35].

Similar to the results observed in this study, treatment of *Leishmania braziliensis* promastigotes with the IC_50_ value of MDL28170 led to a network rupture in the *trans*-Golgi region. In addition, the calpain inhibitor promoted the appearance of diffused concentric membranar structures in the cytoplasm and multivesicular bodies, as well as the formation of blebs in the plasma and flagellar membrane, indicating membrane detachment. Altogether, these ultrastructural changes also suggested an autophagic process induced by MDL28170 in *L. braziliensis* [36]. Previous studies have also reported that the Golgi complex may be a target of MDL28170 in other trypanosomatid species. In *T. cruzi*, ultrastructural analysis of epimastigote forms treated with the IC_50_ value of MDL28170 showed alterations in the Golgi complex as well as severe effects on reservosomes, such that these organelles presented washed-out appearance with a loss of electron density and complete rupture of their membranes [37]. In *Phytomonas serpens*, a tomato parasite related to *Leishmania* spp., the treatment with MDL28170 promoted the rupture of the *trans*-Golgi region with peripheral dilation of cisternae, in addition to mitochondrial swelling with the presence of membranar structures and scarce cristae in the organelle matrix; in addition, dilation of the flagellar pocket was also observed, where several microvesicles were found [38]. The Golgi complex is an essential organelle of the secretory pathway, formed by a stack of interconnected cisterns responsible for processing proteins and lipids and subsequently directing them to the correct intracellular compartment. The *cis*-Golgi cistern receives vesicles from the endoplasmic reticulum, while the *trans*-Golgi cistern is responsible for releasing the vesicles and promoting their final destination [39]. In conclusion, MDL28170 is capable of affecting the *trans*-Golgi network in different species of trypanosomatids, compromising the destination of the substances produced, with consequent deficiency in the composition of the different cellular compartments, affecting the viability of these parasites.

In this study, the deleterious effects of the calpain inhibitor MDL28170 on the viability and ultrastructure of axenic amastigote forms of *L. amazonensis* were investigated. These data made clear that the action of the calpain inhibitor occurs directly on the amastigote form, independently of the reduced ability of the parasite to interact with the host cell. The present manuscript is the first step in the characterization of the mechanisms of action of this molecule in amastigote cells, and experiments are in progress to reach this goal. Overall, the results presented herein may contribute to the further investigation of the mechanisms of action of calpain inhibitors in trypanosomatids and extends the investigation into the use of calpain inhibitors with potential activity in the chemotherapy of leishmaniasis.

## Data Availability

The original contributions presented in the study are included in the article, further inquiries can be directed to the corresponding authors.
